# Report of a semi-branched stent-graft to treat a type 1a endoleak after failed EVAR

**DOI:** 10.1186/s42155-024-00448-4

**Published:** 2024-04-20

**Authors:** Ozan Yazar, ChunYu Wong, Pieter Bartholomeus Salemans, Chrissy van Wely, Ruben Nouwens, Bart van Grinsven, Lee Hans Bouwman

**Affiliations:** 1 Department of Vascular Surgery, Zuyderland Medical Center, Heerlen, The Netherlands; 2 Procurement Department, Zuyderland Medical Center, Heerlen, The Netherlands; 3 Department of Sensor Engineering, Faculty of Science and Engineering, Maastricht University, Maastricht, The Netherlands; 4 Department of Clinical Engineering, Faculty of Science and Engineering, Maastricht University, Maastricht, The Netherlands

**Keywords:** Abdominal Aortic Aneurysm (AAA), Endovascular Aneurysm Repair (EVAR), Branched EVAR (bEVAR), Fenestrated EVAR (fEVAR), Semi-branched EVAR (sbEVAR), Inner-branched EVAR (ibEVAR)

## Abstract

**Background:**

Endovascular techniques are advancing with the change of treatment paradigm for abdominal aortic aneurysms. Fenestrated EVAR (fEVAR) and branched EVAR (bEVAR) are used for complex aortic aneurysm repair. Both fEVAR and bEVAR have their own advantages and disadvantages. Semi-branches are a new feature that attempt to combine the advantages of both fEVAR and bEVAR.

**Technique:**

We describe the use of a 4-vessel semi-branched EVAR in a failed EVAR case with a type 1a endoleak.

**Conclusion:**

The novel feature of semi-branches in custom-made EVAR devices in endovascular aortic treatment following failed EVAR appear to be a feasible option.

**Supplementary Information:**

The online version contains supplementary material available at 10.1186/s42155-024-00448-4.

## Introduction

Since the introduction of endovascular management of abdominal aortic aneurysms in 1990, Endovascular Aneurysm Repair (EVAR) has become the first line treatment in a large part of the world [1]. EVAR accounts for approximately 70% of all abdominal aortic aneurysms (AAA) in Europe. However, as it has well been acknowledged, EVAR treatment has limited anatomic properties such as infra-renal neck length, angulations, vascular access, thrombus in landing zones and calcification. These properties are defined in the instruction for use (IFU) of the EVAR stent-grafts. Performing EVAR procedures outside of IFU can result in incomplete exclusion of the aneurysm or early failures [2]. Type 1a endoleaks are life-threatening due to the risk of rupture and should be treated promptly.

Great technical advances have been made to expand the endovascular treatment of AAA and thoracoabdominal aortic aneurysms (TAAA). In 1996 fenestrated EVAR (fEVAR) was introduced, allowing treatment of AAA to extend above the renal arteries [3]. In 2001 the endovascular arsenal was expanded by branched EVAR (bEVAR) [4]. Both fEVAR and bEVAR have unique advantages and disadvantages allowing both treatment options to be complementary to each other. Advantages of fEVAR are the ability to treat narrow aortas, have less proximal aortic coverage and treat transverse offset target vessels. Disadvantages of fEVAR are that there is no real sealing or fixation of the bridging stents, the deployment must be meticulous and is not forgiving and steep offset target vessels may result in kinking of bridging stents. Advantages of bEVAR are that large aneurysms can be treated, there is a proper sealing and fixation to the bridging stents and placement is relatively forgiving. The drawbacks of bEVAR are that there is more proximal coverage of the aorta, it cannot be used in a narrow aorta and branches can get squashed in kinked anatomy.

In an attempt to combine the advantages of both fEVAR and bEVAR, inner branched EVAR (ibEVAR) was developed in 2001 [4]. A reduction of diameter is attained by internalizing the branches of the main body of the stent instead of placing branches in the outer part of the stent graft. This results in the ability to treat a narrow aorta. As the device is branched, proper sealing is provided for bridging stents and placement of the device is forgiving. Finally, less proximal aorta is covered as compared to bEVAR.

In the bEVAR evolution semi-branches have been introduced as a new feature and meeting all legal criteria a full market release was done in 2023. Semi-branches are inner branches with a reduced length of 6 mm. The branch is sown in the main body as a shortened inner branch and fixated with 3 stiches proximally. The outlet is oval shaped with a variable length and diameter, varying from 10 to 12 mm and 6 to 8 mm respectively. While the structural configuration of fEVAR is mimicked by using semi-branches, a seal and fixation for the bridging stent is provided. Furthermore, target vessel canulation is facilitated as the outlet of the semi-branched EVAR (sbEVAR) is oval and there is a larger freedom of positioning the bridging stent. Since semi-branches are a relatively novel feature, there is few literature describing its outcomes. The available literature describes adding semi-branches as helpful in pathologies where fenestrations are not suitable due to kinked aortas or pathologies with a small aortic diameter lacking space for inner or outer branches [5].

**Fig. 1:**
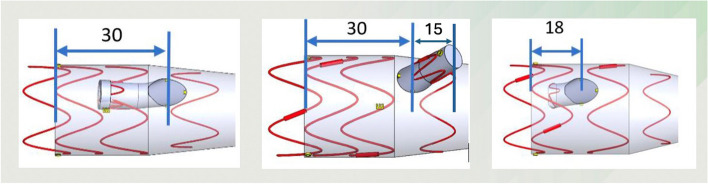
Minimal proximal aorta coverage (in mm) of inner branched EVAR, outer branched EVAR and semi-branched EVAR

Perhaps the most important feature of the semi-branches is that the proximal aortic coverage of the aorta is reduced. A regular (outer) bEVAR has a minimum proximal aortic coverage above the center of the coeliac trunk outlet of 45 mm and a regular (inner) bEVAR of 30 mm. The sbEVAR has a proximal aortic coverage of as little as 18 mm subsequently reducing the risk of spinal ischemia (Fig. 1). The proximal fixation of the stentgraft is obtained by a Nitinol topstent without hooks.

In this paper we describe the semi-branch technology usage in EVAR failure due to progression of disease.

## Technique

**Fig. 2:**
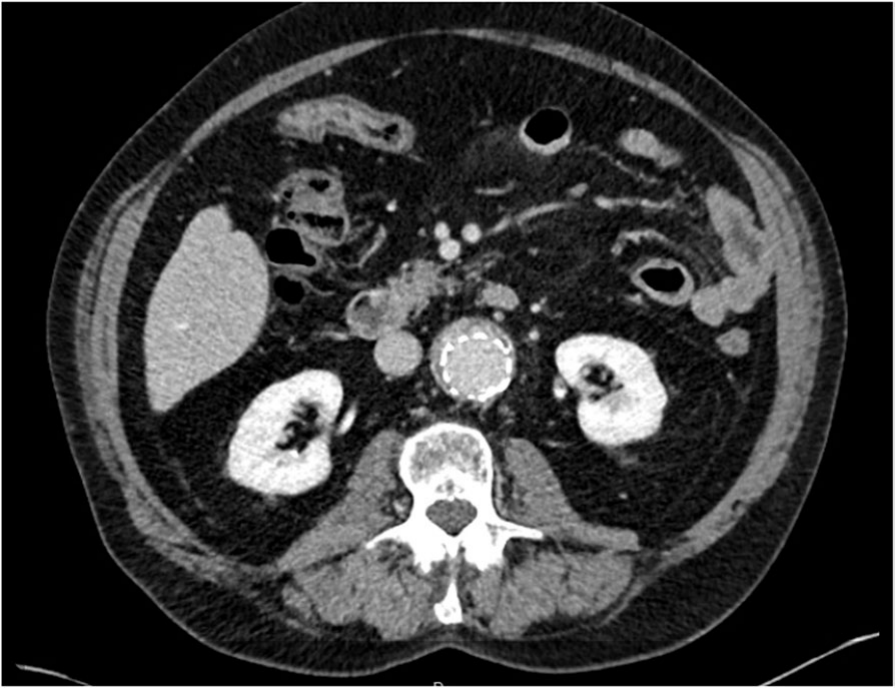
Patients Computed Tomography Angiography showing a type 1 endoleak

An 85-year-old patient received an EVAR in 2019 for a 53 mm AAA. The aneurysm had an infra-renal neck length of 13 mm and was treated with an Endurant stent-graft (Medtronic Santa Rosa, CA, United States of America) within the IFU. Persistent growth in diameter of the AAA was observed on doppler ultrasound in the follow-up visit. Computed Tomography Angiography (CTA) confirmed AAA growth of 6 mm compared to preoperative CTA and showed an endoleak. Primarily the endoleak was classified as a type 2 originating from the inferior mesenteric artery (IMA). Coiling of the IMA was successfully performed. During the follow-up CTA a persistent endoleak was revealed and was classified as type 1a (Fig. 2).

**Fig. 3:**
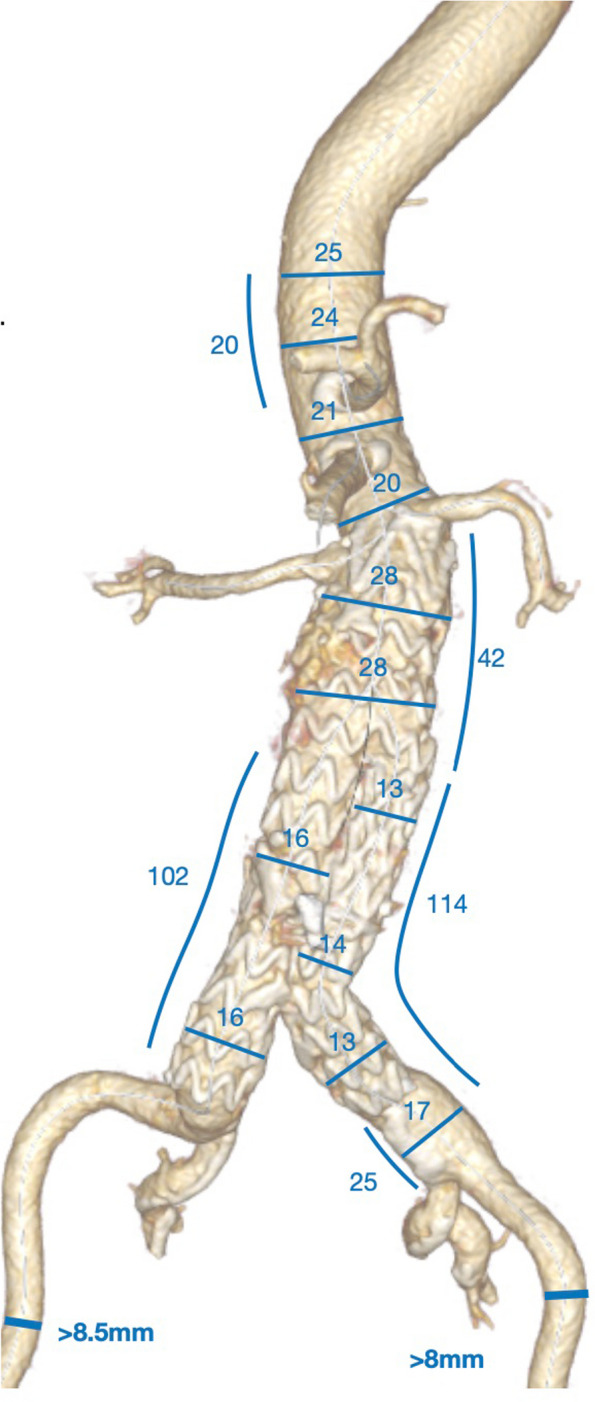
3D reconstruction for initial anatomy evaluation

**Fig. 4:**
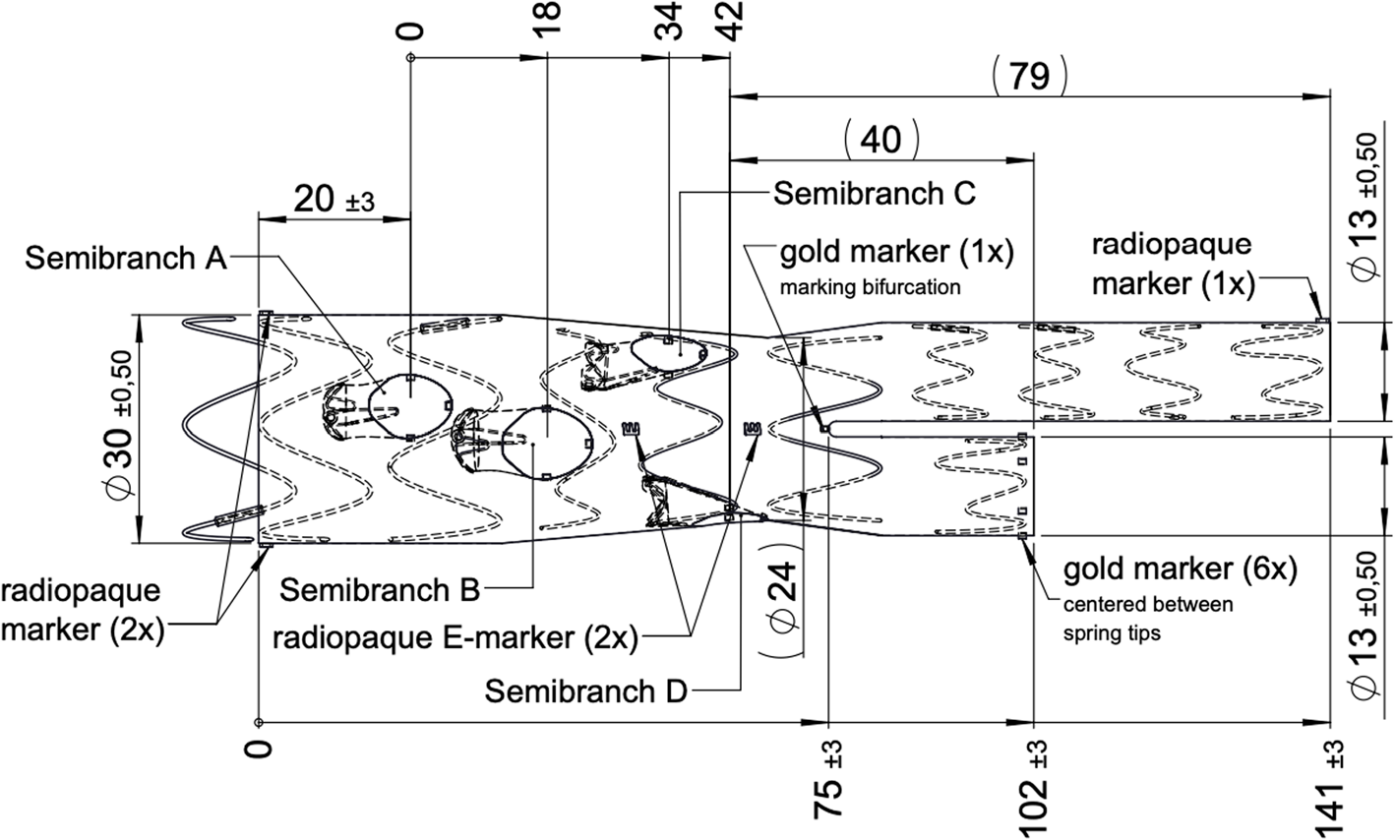
Final stent design

**Fig. 5:**
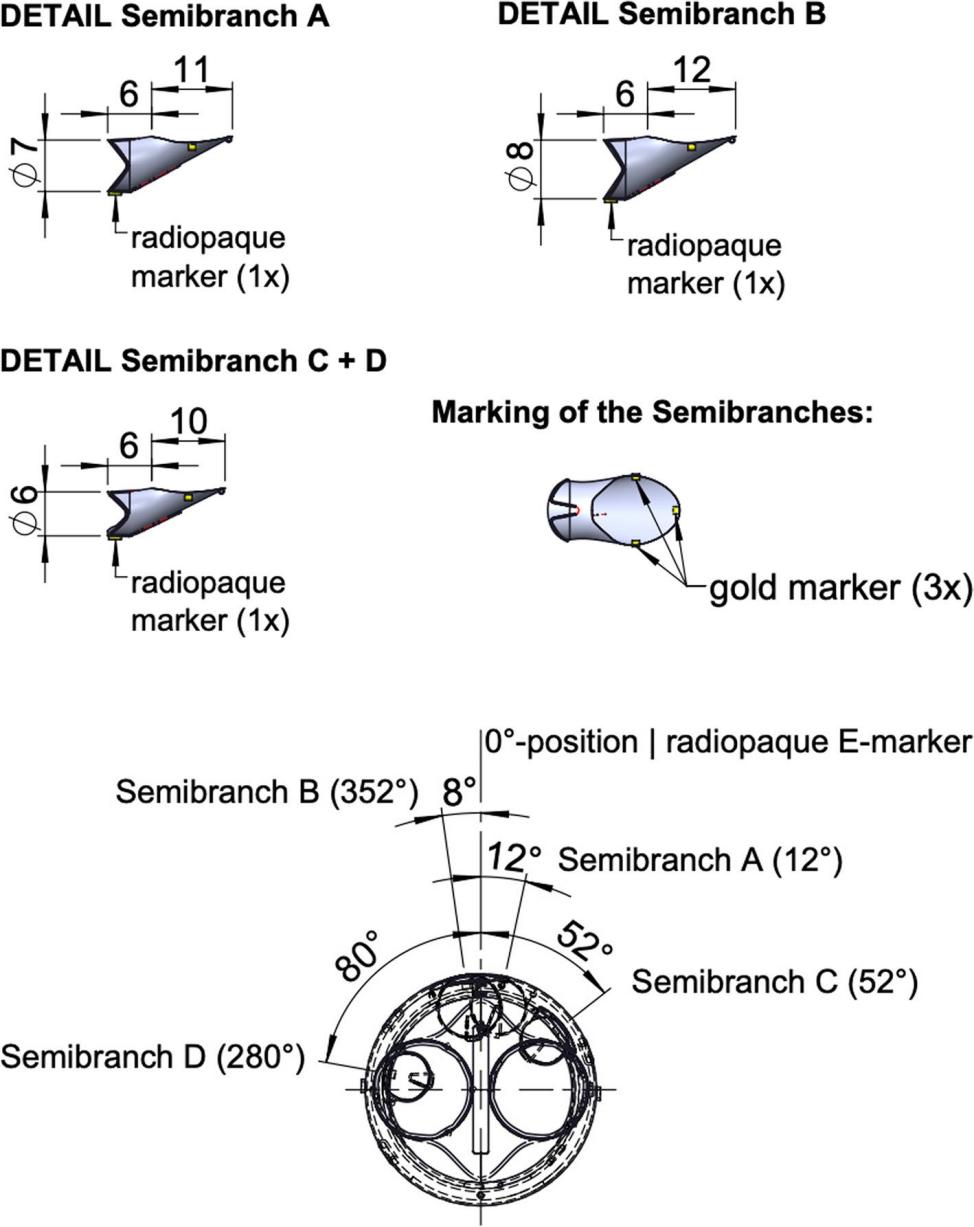
Design semi-branches and inner view of the stent design

A custom made 4-vessel sbEVAR was designed and produced by Artivion GmbH (Kennesaw, GA, United States of America). To fully exclude the type 1a endoleak, full realignment of the EVAR was chosen as opposed to proximal extension by a branched cuff. Initial evaluation of the anatomy is shown in Fig. 3 and the final stent design is shown in Figs. 4 and 5. At 22 mm and 39 mm above the mid coeliac trunk there were pairs of lumbar arteries. In order to preserve as many lumbar arteries as possible, proximal coverage of the aorta was minimalized to a total length of 20 mm proximal to the midst of the coeliac trunk outlet using semi-branches. With an inner bEVAR the pair at 22 mm would have been lost and with an outer bEVAR pairs at 22 and 39mm would have been lost. A partial deployment technique was performed as the suprarenal aortic diameter was as narrow as 20 mm at certain parts. This required cannulation of the target vessels from above.

This case was performed in a hybrid operating suite under general anesthesia and systematic heparinization. Percutaneous access was obtained by ultrasound guided placement of two 7 French sheaths in both common femoral arteries. A cutdown was performed of the left axillary artery and a 7 French sheath was placed. A Glidewire Advantage (Terumo, Inchinnan, Scotland) guidewire was placed in the abdominal aorta via the axillary artery. The 7 French sheath was replaced by a 45 cm 12 French sheath, acting as a shuttle sheath. Through the 12 French sheath a 70 cm 8 French sheath was advanced, and an angiography was performed by using a universal flush catheter (UF).

**Fig. 6:**
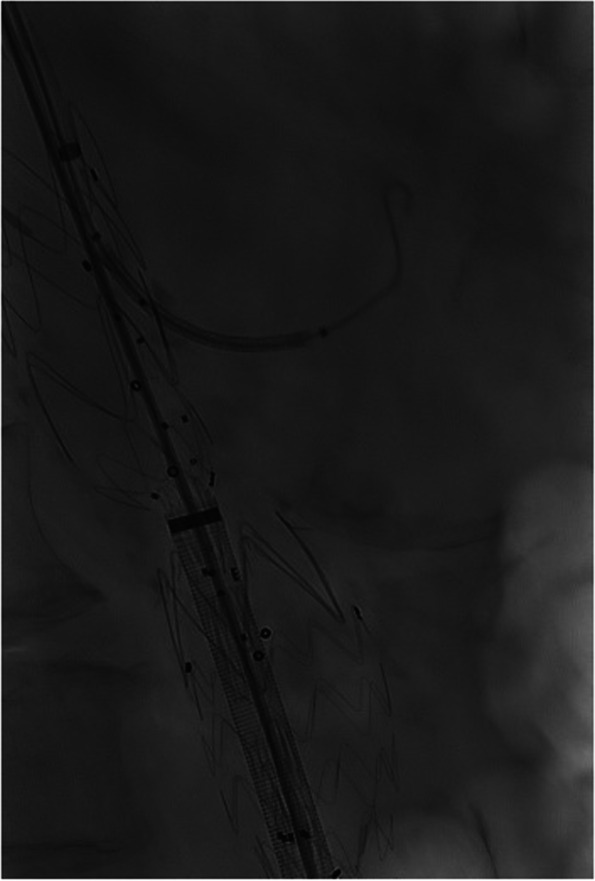
Canulation and stenting of the coeliac trunc

Via the left groin a 0.035″ Terumo guidewire with an UF catheter was advanced into the aortic arch. The guidewire was exchanged for a stiff Lunderquist guidewire (Cook Medical, Bloomington, Indiana, United States of America) and the UF was removed. The custom-made 4 vessel sbEVAR was advanced over the stiff guidewire. Angiography was performed via the UF catheter placed via the axillary access. The sbEVAR was placed upon alignment on the renal arteries. The sbEVAR was partially deployed exposing the inlet and outlet of the coeliac trunk (CT). From above, using a vertebral catheter both the CT-branch and the CT were canulated (Fig. 6). The guidewire was exchanged for a Rosen-wire (Cook Medical) and a 7 × 37 mm E-Ventus covered stent (Jotec/Artivion, Erkelenz, Germany) was placed. The stent was placed with the proximal part 5 mm above the inlet of the branch and was flared by over dilating to burst pressure (8 mm).

The sbEVAR was further deployed, exposing the inlet and outlet of the superior mesenteric artery (SMA). The SMA was cannulated and stented using a 8 × 37 mm E-Ventus stent (Jotec/Artivion) and flared proximally (9 mm). Finally, the sbEVAR was fully deployed exposing both the left and right renal artery branches. The left and right arteries were cannulated with E-Ventus stents (respectively 6 × 38 mm and 6 × 58 mm) (Jotec/Artivion). Both proximal parts were flared up to 8 mm. The sbEVAR was extended distally with a 1519L10 limb (Jotec/Artivion) on both sides, preserving both hypogastric arteries. Final angiography showed patent target vessels and no sign of endoleak. Percutaneous punctures were closed using the Proglide closing system (Abbott Vascular, Santa Clara, CA, United States of America). The axillary access was closed surgically.

**Fig. 7:**
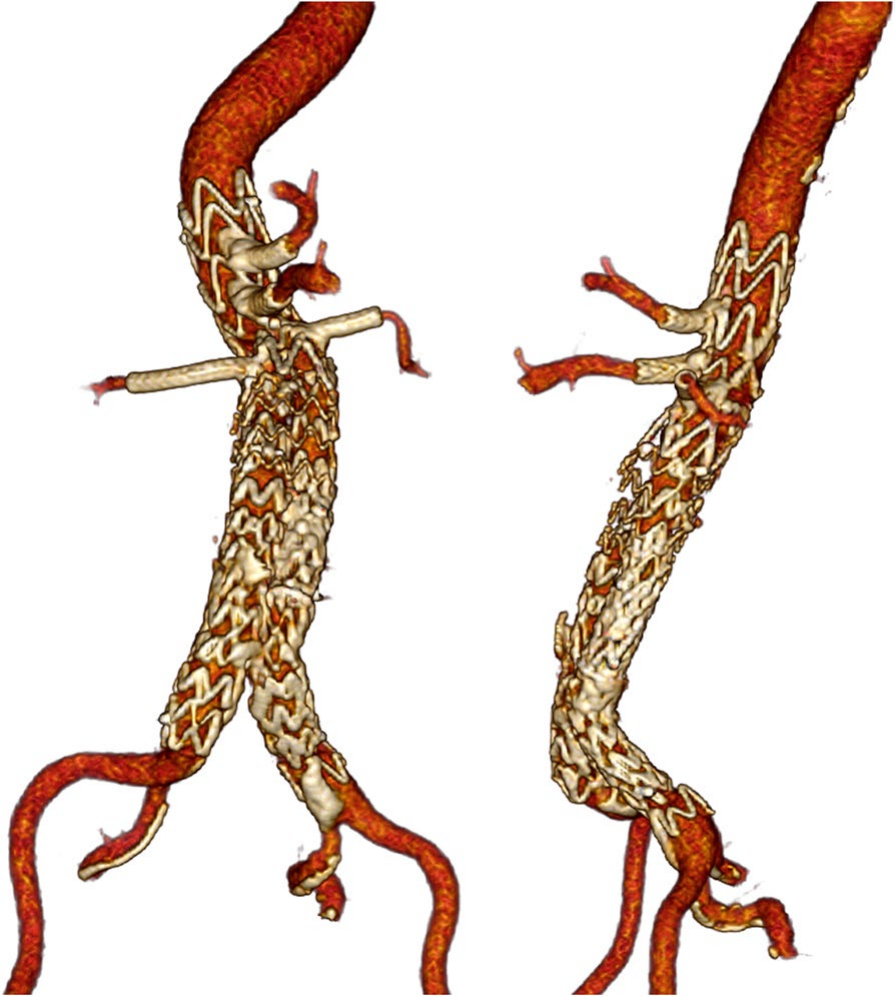
Computed Tomography Angiography 3D reconstruction

Post operative recovery of the patient was uneventful. Follow-up CTA at 6 weeks showed patent target vessels and no signs of endoleak (Fig. 7). Furthermore, all lumbar arteries (22 mm and 39 mm above mid coeliac trunk) were patent.

## Discussion

With the change of treatment paradigm for infrarenal abdominal aortic aneurysms, failed EVAR continues to be a growing and challenging issue. Various custom-made devices such as fEVAR, bEVAR and physician modified devices have been used to treat patients [6–8]. Individual anatomy as well as the presence of suprarenal fixation of the primary EVAR can increase technical challenges even more. It is therefore of great importance that additional options are developed to expand the arsenal of technical solutions. The ability to combine various features within one stent graft increases endovascular treatment possibilities. The option of including semi-branches combines the advantages of fEVAR and bEVAR. The inner branches are shortened, the structural configuration of fEVAR is mimicked while a seal and fixation for the bridging stent is provided. Additionally, proximal aortic coverage can be reduced. This case has demonstrated that the option of including semi-branches may be helpful in designing a custom-made solution. Although the advantages of reduction of aortic coverage appears to be clear, the long-term results are not known. Reducing the sealing zone of bridging stents may result in type 1c endoleak. Benchtop research with regards to pullout strength of bridging stent, sealing efficiency and durability of bridging stents are required. Furthermore long-term follow-up of the sbEVAR is essential.

## Conclusion

The feature of semi-branches in custom-made EVAR in endovascular aortic treatment following failed EVAR appears to be a feasible option. However mid and long-term results with regards to sealing and endoleak are lacking. Data on the semi-branched EVAR system and need to be studied in a larger patient cohort with.

### Supplementary Information

**Supplementary Material 1.**

## Data Availability

Not applicable.
